# Primary health care and the climate crisis

**DOI:** 10.2471/BLT.20.252882

**Published:** 2020-09-28

**Authors:** Sowmya Kadandale, Robert Marten, Sarah L Dalglish, Dheepa Rajan, David B Hipgrave

**Affiliations:** a United Nations Children’s Fund, World Trade Centre 2 (22nd Floor), Jalan Jenderal Sudirman Kav. 29-31, Jakarta 12920, Indonesia.; b Alliance for Health Policy and Systems Research, World Health Organization, Geneva, Switzerland.; c Department of International Health, Johns Hopkins School of Public Health, Baltimore, United States of America (USA).; d Department for Health Systems Governance and Financing, World Health Organization, Geneva, Switzerland.; e United Nations Children’s Fund, New York, USA.

The health community recognizes the climate crisis as an existential threat to humanity and human health,^1^ requiring immediate and effective action across all sectors. However, global-level policy guidance reveals a disconnect between primary health care and climate; most political declarations, reports and resolutions for primary health care make only cursory references to the climate crisis, mentioning its implications for health but not linking them. Given that primary health care should be the entry point for the population’s interaction with the health system, it is alarming that ongoing efforts to revitalize primary health care fail to adequately consider climate action, both in terms of mitigation and adaptation. In this paper, we examine this disconnect, elaborate on its implications and offer recommendations for policy-makers to ensure an effective primary health care–climate crisis nexus.

We argue that the link between climate change and the three components of primary health care (that is, comprehensive primary care and public health interventions; multisectoral action; and community empowerment) must be better connected. For example, excessive greenhouse gas emissions lead to more polluted air, with implications for acute and chronic respiratory disease; droughts, melting glaciers and climate-related natural disasters such as fires are already impacting food production. The impact of climate change on livelihoods is forecast to cause large-scale societal disruptions and exacerbated inequities, and result in population migration, poverty and poorer health. As such, the climate crisis will have a major effect on both health and life expectancy.

Among the health plans of the Group of Twenty, only two countries refer to climate change: France recognizes that global warming will contribute to an increase in vector-borne diseases and Indonesia acknowledges the need for subnational strategies to adapt to climate-related health consequences. No plans include an analysis of climate change or its impact on the ability of countries to deliver primary health care. The climate national adaptation plans of these countries (with the exception of the European Union, not included in the analysis) address the primary health-care component on multisectoral action, particularly with reference to water, sanitation and hygiene, and nutrition and agricultural interventions; however, the other two components are largely ignored. South Africa called for scaling up of existing public health interventions in response to the climate crisis, and India and the United States of America included specific plans for community engagement (for example, through community research and building community resilience). No other climate plans discuss the link to or impact of the climate crisis on primary care services or essential public health functions, or the potential contribution of community engagement to climate change mitigation and adaptation.

This context illustrates the need for the primary health care and the climate discourses to converge in a deeper, more meaningful way. While climate plans often consider predicted health effects, they less frequently consider the need to adapt primary care services or the more explicit engagement of communities in decisions on activities that might impact both health and the climate crisis. Conversely, national health plans rarely mention climate change, let alone provide a detailed analysis of how primary health care and the wider health system must evolve to meet the challenge from a systems perspective as well as address changing health needs.

These omissions are alarming, given the multiple pathways through which the climate crisis is affecting population health needs and outcomes. We review each of the three components of primary health care and consider linkages to jointly address such care and the climate crisis.

## Primary care and public health interventions 

Primary care is the first point of contact intended to provide comprehensive and coordinated health services; growing climate-related stresses could create breaking-point conditions on such care and affect key parts of primary care services such as facilities, human resources and supply chains.^2^ The health response to the climate crisis will be delivered by primary health-care workers, who are equally affected by climate-related threats to housing, food prices, exposure to extreme weather events and vector- and waterborne diseases.^3^ Primary care providers must contribute to both climate change adaptation and mitigation via preventive and promotive health measures (such as healthy diets) and integrated health services.^4^ For example, in the United Kingdom of Great Britain and Northern Ireland, nurses and their associations across all levels are addressing climate change by raising awareness and calling for greater climate justice as well as advocating for and implementing climate adaptation and mitigation strategies.^5^ The National Health Service recently announced its goal to become net carbon zero by 2040. While professional associations can make positive changes and create awareness, many aspects of climate change adaptation and mitigation can only be addressed by national and subnational policy-makers, for example in the way that health facilities and workers consume energy and food, and use transport and logistics to procure supplies. In terms of mitigation, policy-makers (especially in high-income, high-carbon emitting countries) could move towards local procurement of supplies to reduce the carbon footprint from transport, orient health facility-based food services towards more locally produced plant-based diets and use renewable energy. In terms of adaptation (especially in low- and middle-income countries that are likely to be most affected by climate change), policy-makers must anticipate likely local disruptions, both in terms of health needs and the ability to deliver them. Policy-makers must also plan for these disruptions in every aspect of primary care and public health interventions.

## Multisectoral action

Activities in many non-health sectors such as education and transportation benefit both primary health care and tackling the climate crisis,^6^ particularly in relation to noncommunicable diseases as well as fossil fuels and pollution.^7^ For example, the promotion of healthy diets and investments in sustainable food systems have demonstrated benefits from both the climate and health perspectives, and offer options for identifying food sources that require less resources such as land, water and energy to produce.^8^ Significantly, determinants of health cut across different sectors, including those that have an impact on both health and climate, such as fossil fuel burning for transport, which leads to pollution and in turn affects health outcomes. Although multisectoral action is a key component of primary health care, many health systems struggle with delivering or influencing interventions that span multiple sectors. As such, the critical action is not designing interventions, but rather strengthening governance structures across sectors to meet the challenges of the climate crisis.

## Community empowerment

The primary health-care level is where health and community action intersect most closely, with health workers and managers based in primary care facilities going into communities for outreach programmes and health awareness campaigns. This community–health interface provides an entry point contributing to social capital and resilience to the climate crisis.^9^ Ensuring responsive public health and preventive and curative health services requires community empowerment and mobilization. The ongoing coronavirus disease 2019 pandemic demonstrates the importance of community engagement for effective public health action.^10^ As well, the youth-led global movement to fight climate change and force policy-makers to recognize the climate emergency is growing. Primary health care thus represents an opportunity to capture synergies and strengthen and mobilize communities to deliver the public goods valued by both individuals and societies: health today and for future generations. Many social participation mechanisms for health currently exist, providing potential to foster grassroots engagement on the climate crisis more holistically. Such efforts provide opportunities to operationalize and capitalize on the connections between primary health care and the climate crisis.

The World Health Organization’s vision for primary health care raises broader questions on responsive, efficient and cost-effective service delivery as well as equitable outcomes. Shifting disease patterns and the other effects of climate change disproportionately affect low-income countries and vulnerable populations, as well as small island states, many of which contribute least to global carbon emissions.^11^ The inequities fuelled by the climate crisis and its associated environmental consequences have dire public health consequences, especially for poor people, older adults, children, racial and ethnic minorities, and other vulnerable groups. Additionally, the climate crisis threatens to exacerbate migration as well as gender-based health disparities:^12^ large-scale migration will increase because of resource scarcity and extreme weather events, and women face higher risks and greater burdens from climate change. Primary health care must be purposefully adapted to counter these worsening and unequally distributed problems. Moreover, high-income countries must do more to reduce their share of carbon pollution and help stem the worst effects of climate change. More positively, primary health care adaptation and improvement offer opportunities to both improve population health and well-being, and to empower populations to catalyse positive changes to both health and the climate crisis.

We present some potential recommendations for policy-makers to stimulate thinking on how these issues could be considered (Box 1).

Box 1 **Potential recommendations for policy-makers**

**At the country level:**

• Revise existing health plans to include a robust situation analysis of the climate landscape and health implications, designing primary health care-based approaches to address the gaps;

• Ensure the heath sector is actively engaged in climate crisis decision-making;

• Include primary health care-based approaches across the life course and analysis of interventions for the climate crisis in national plans, commitments and budget allocations;

• Lead by example, ensuring that health systems are not only net zero (that is, that they do not contribute to the climate crisis) but are green leaders across government sectors and within communities; and

• Empower key stakeholders across sectors to work at the local (subnational) levels to engage communities to design primary health care-based solutions aimed at tackling the climate-related impacts.

**At the global/regional levels:**

• Provide normative guidance on primary health care that directly addresses threats to populations, communities and systems posed by the climate crisis;

• Ensure that primary health care dialogues, declarations and resolutions do not overlook key climate-related issues, but rather explicitly work to accelerate decarbonization and the move away from fossil fuels;

• Collate and disseminate best practices from countries that have successfully integrated climate adaptation and mitigation policies into their primary health-care systems and services, keeping updated, centralized, easily accessible global databases on climate plans, health plans and primary health care strategies to profile and highlight best practices;

• Continue to build a focus on primary health care and climate-related issues through the *Global action plan for healthy lives and well-being for all*, particularly the accelerator on primary health care, ensuring their linkage and integration; and

• Support countries in holistically applying global guidance on primary health care and climate-related issues.

To achieve the health-related sustainable development goal, greater convergence between leadership and community action on primary health care and the climate crisis is required. Such a linkage would provide services that are adapted to populations’ evolving needs in the context of the climate crisis, transform how primary health care is delivered in the face of an evolving existential threat, reduce the health sector’s contribution to the emergency, and work across sectors and with communities to provide more unified and effective strategies to promote health and fight climate change. We believe that primary health care and the climate crisis cannot be dealt with separately. 

## References

[1] Climate change is the greatest threat to human health in history. Bethesda: Health Affairs; 2018. Available from: https://www.healthaffairs.org/do/10.1377/hblog20181218.278288/full/ [cited 2020 Aug 3].

[2] Salas RN, Jha AK. Climate change threatens the achievement of effective universal healthcare. BMJ. 2019 9 23;366:l5302. 10.1136/bmj.l5302 31548271 PMC6753637

[3] Haines A, Scheelbeek P. The health case for urgent action on climate change. BMJ. 2020 3 30;368:m1103. 10.1136/bmj.m1103 32229547 PMC7190277

[4] Xie E, de Barros EF, Abelsohn A, Stein AT, Haines A. Challenges and opportunities in planetary health for primary care providers. Lancet Planet Health. 2018 5;2(5):e185–7. 10.1016/S2542-5196(18)30055-X 29709275

[5] Nicholas PK, Breakey S. Climate change, climate justice, and environmental health: implications for the nursing profession. J Nurs Scholarsh. 2017 11;49(6):606–16. 10.1111/jnu.12326 28749596

[6] Gould S, Rudolph L. Challenges and opportunities for advancing work on climate change and public health. Int J Environ Res Public Health. 2015 12 9;12(12):15649–72. 10.3390/ijerph121215010 26690194 PMC4690946

[7] Climate change, air pollution and noncommunicable diseases. Geneva: World Health Organization; 2019. Available from: http://www.who.int/bulletin/volumes/97/2/18-224295/en/ [cited 2020 Jun 6]. 10.2471/BLT.18.224295 PMC6357572 30728622

[8] Aleksandrowicz L, Green R, Joy EJM, Smith P, Haines A. The impacts of dietary change on greenhouse gas emissions, land use, water use, and health: a systematic review. PLoS One. 2016 11 3;11(11):e0165797. 10.1371/journal.pone.0165797 27812156 PMC5094759

[9] Ebi KL, Semenza JC. Community-based adaptation to the health impacts of climate change. Am J Prev Med. 2008 11;35(5):501–7. 10.1016/j.amepre.2008.08.018 18929976

[10] Marston C, Renedo A, Miles S. Community participation is crucial in a pandemic. Lancet. 2020 5 30;395(10238):1676–8. 10.1016/S0140-6736(20)31054-0 32380042 PMC7198202

[11] Bennett CM, Friel S. Impacts of climate change on inequities in child health. Children (Basel). 2014 12 3;1(3):461–73. 10.3390/children1030461 27417491 PMC4928733

[12] Sorensen C, Murray V, Lemery J, Balbus J. Climate change and women’s health: impacts and policy directions. PLoS Med. 2018 7 10;15(7):e1002603. 10.1371/journal.pmed.1002603 29990343 PMC6038986

